# Triad pattern algorithm for predicting strong promoter candidates in bacterial genomes

**DOI:** 10.1186/1471-2105-9-233

**Published:** 2008-05-09

**Authors:** Michael Dekhtyar, Amelie Morin, Vehary Sakanyan

**Affiliations:** 1 University of Tver, 33 Jelyabova, 170000 Tver, Russia; 2 Laboratoire de Biotechnologie, UMR CNRS 6204, Université de Nantes, 2 rue de la Houssinière, 44322 Nantes, France; 3 ProtNeteomix, 2 rue de la Houssinière, 44322 Nantes, France

## Abstract

**Background:**

Bacterial promoters, which increase the efficiency of gene expression, differ from other promoters by several characteristics. This difference, not yet widely exploited in bioinformatics, looks promising for the development of relevant computational tools to search for strong promoters in bacterial genomes.

**Results:**

We describe a new triad pattern algorithm that predicts strong promoter candidates in annotated bacterial genomes by matching specific patterns for the group I σ^70 ^factors of *Escherichia coli *RNA polymerase. It detects promoter-specific motifs by consecutively matching three patterns, consisting of an UP-element, required for interaction with the α subunit, and then optimally-separated patterns of -35 and -10 boxes, required for interaction with the σ^70 ^subunit of RNA polymerase. Analysis of 43 bacterial genomes revealed that the frequency of candidate sequences depends on the A+T content of the DNA under examination. The accuracy of *in silico *prediction was experimentally validated for the genome of a hyperthermophilic bacterium, *Thermotoga maritima*, by applying a cell-free expression assay using the predicted strong promoters. In this organism, the strong promoters govern genes for translation, energy metabolism, transport, cell movement, and other as-yet unidentified functions.

**Conclusion:**

The triad pattern algorithm developed for predicting strong bacterial promoters is well suited for analyzing bacterial genomes with an A+T content of less than 62%. This computational tool opens new prospects for investigating global gene expression, and individual strong promoters in bacteria of medical and/or economic significance.

## Background

Efficient promoter recognition is crucial in the synthesis of the gene-encoded products required by bacteria to allow them to grow rapidly and to adapt to different environmental conditions. The general architecture and protein-DNA interaction interfaces appear to be conserved in RNA polymerases of different bacteria, to judge by a comparison of the resolved structures of the multi-subunit protein or its subunits [1]. This structural information suggests that the principles of DNA recognition by RNA polymerases are universal, and this constitutes a basis for *in silico *prediction of promoters that are recognized by families of sigma factors. Research in bioinformatics has developed approximate matching methods to detect conserved sequences in nucleic acids [2-5], including promoter-specific sequences that are invaluable in helping to elucidate the overall organization of transcriptional signals and regulatory circuits in evolutionarily distant bacteria [6-14]. Most promoter prediction programs so far proposed use statistical or motif-based methods, and take into consideration what is known about experimentally defined promoter architectures, and extract conserved sequences from the genomes under analysis. Attempts have been made to improve promoter prediction by introducing statistical mechanical methods to measure the stress-induced destabilization or bendability of the duplex DNA region located upstream of the transcription initiation site required for the local dissociation of strands to start mRNA synthesis [15-17]. The steady increase in the number of sequenced bacterial genomes of medical and economic significance means that there is an increasing need for computational tools to predict promoters, especially those responsible for high-level gene expression in organisms, of which there has been little experimental investigation.

Many housekeeping genes in *Escherichia coli *are transcribed from promoters possessing the recognition elements referred to as -35 and -10 sites (boxes), for which the TTGACA and TATAAT consensi, respectively, have been identified by compiling characterized RNA polymerase-binding regions in the DNA [18,19]. The -35 and -10 sites, which are separated from each other by a 15–20-bp spacer [20], are specifically recognized by Eσ^70 ^RNA polymerase, an RNA polymerase holoenzyme bearing the group I σ^70 ^factor [21]. Experimental data have shown that high transcription rates of genes correlate with the level of conservation of three promoter parameters, with the consensus -35 and -10 hexanucleotide boxes, and with the 17 ± 1-bp spacer separating them [22]. This fact has been widely used to construct vectors for protein overexpression in bacterial cells [23].

However, the strength of strong promoters also depends on a fourth parameter, an AT-rich UP element 17–20 bp length, which is located just upstream of the -35 site, and which is recognized by the α subunit of Eσ^70 ^RNA polymerase, which was first discovered for ribosomal RNA promoters [24]. The C-terminal domain of this subunit binds both to the UP element and to transcription regulation proteins, whereas the N-terminal domain makes contact with other subunits during the assembly of RNA polymerase [25]. A 17-bp consensus 5'AAAWWTWTTTTNNNAAA (where W is A or T, and N can be any of the four bases) has been identified for the UP element by analyzing the patterns, selected by the SELEX method, which mediate increases of between 10- and 300-fold in gene expression in *E. coli *cells [26-28]. Two preferred sub-sites have been identified within the UP element. They are centered approximately at the -42 and the -52 positions respectively, and appear to be specifically recognized by one or two monomers of a dimeric α subunit in the RNA polymerase.

It is noteworthy, that a virtual analysis of patterns located upstream from the consensus -35 had since long time suggested their functional significance [3]. The sequences reminiscent of UP element have been detected in the *E. coli *genome by the algorithms PWM [29] and PlatPram [30]. A software-based (GCG, version 9.0) dissection of regions located upstream of the *E. coli *promoters had made it possible to detect putative promoters with ≤ 4 mismatches in the full UP element consensus [28]. Several UP elements have also been visually identified, and characterized by their ability to direct high level gene expression *in vivo *or *in vitro *in *Bacillus subtilis *[31], *Geobacillus *(formerly *Bacillus*) *stearothermophilus *[32] and *Vibrio natrigens *[33]. Recently, a comparative analysis of Eσ^70^-specific promoter and non-promoter regions indicated that upstream regions of *E. coli *ribosomal and T4 phage early promoters possess electrostatic elements that could be responsible for modulating promoter activities due to ADP-ribosylation of RNA polymerase α subunit [34]. However, no specific algorithms have yet been proposed to detect strong promoters in bacterial genomes, and so this remains an important task for genomic and proteomic research in microbiology.

In this study, we have developed a triad pattern algorithm that detects strong promoter candidates composed of a UP-element, and two consensi, -35 and -10 boxes, which are optimally distanced from each other. All four parameters are required for efficient DNA recognition, and the initiation of mRNA synthesis by an Eσ^70^-like RNA polymerase. The data presented indicate that the frequency of strong promoters is a function of the A+T content of the corresponding genomes. The proposed prediction program is flexible, and can be modified by users to modulate the search stringency criteria depending on the promoter features of the genome under analysis. The accuracy of detection has been experimentally validated for putative strong promoters predicted in a hyperthermophilic bacterium *Thermotoga maritima*.

## Implementation

### Overview of the approach

The promoter activity in cells is determined by regulatory proteins (activators and repressors) that can recognize overlapping sequences specific for Eσ^70 ^RNA polymerase sites, and thereby mask the true promoter strength. In addition, almost 20% of *E. coli *RNA polymerase Eσ^70^-specific promoters possess an extended -10 sequence that might compensate for the absence of a clear -35 site [35]. Different prediction programs based on statistical and motif-searching approaches have been developed to detect a variety of binding sites in DNA, and both position-specific weight matrices [36] and hidden Markov models [37] have been used to improve the accuracy of the prediction of promoter sequences in bacterial genomes [38-40]. These programs usually detect hexanucleotide dyad patterns of RNA polymerase-promoter binding sites, such as -35 and -10 boxes, and none of them is free of false-positives, which correspond to similar, non-promoter sequences in bacterial genomes [for a review, see [41]].

In this study, we exploited the strengths of the "triad pattern" approach to develop an algorithm able to detect strong promoters by matching three nucleotide sequences recognized by the σ^70 ^and α subunits of bacterial RNA polymerase. Theoretically, the presence of a UP element may not be essential for relatively strong promoter activity if two -35 and -10 boxes are well conserved and optimally distanced. Similarly, the presence of a well conserved UP element may compensate for a poor -35 box in some promoters. However, it seems very likely that the strongest promoters probably possess all three essential sequences. The specific interaction between the UP element and the α subunit significantly amplifies the association of RNA polymerase with promoter DNA [27,28]. Therefore, to improve the filter to exclude possible false-positive due to short hexanucleotide similar sequences scattered throughout the genome, our algorithm starts by first matching the UP element, and only then identifying the -35 and -10 boxes located further downstream.

### Design of the triad pattern algorithm

We designed an algorithm able to detect the triad nucleotide patterns in bacterial genomes. The core of the algorithm is the FIND_TRIAD procedure, which given an input nucleotide string, *s*, returns the substring *s' *of *s*, which is the best approximation of a given triad pattern of the form *(pat(1),L1)-(l1,l2)-(pat(2),L2)-(d1,d2)-(pat(3),L3)*, where each *pat(i), i = 1,2,3*, is a nucleotide string, *Li *is its length, *l1 *and *l2 *are the minimum and maximum distances respectively between the first and the second patterns, and *d1 *and *d2 *are the minimum and maximum distances respectively between the second and the third patterns. To avoid making a "bad" approximation, three scores *Sc1, Sc2 *and *Sc3 *are used as input parameters for the procedure. The resulting substring, *s'*, can then be represented as (s*pat(1),Ls1)-ls1-(spat(2),Ls2)-ls2- (spat(3),Ls3)*, where each *spat(i), i = 1,2,3*, is a substring of *s *aligned to *pat(i)*, *Lsi *is its length, *ls1 *is the distance between s*pat(1) *and s*pat(2)*, and *ls2 *is the distance between s*pat(2) *and s*pat(3)*. This result for *s' *satisfies the following conditions:

(1) for each *i = 1,2,3 *the similarity score (weight) *Wi *of the match or alignment of *pat(i) *and *spat(i) *is not less than *Sci *(or the number of "mismatches" does not exceed *(Li - Sci)*);

(2) *(l1 ≤ ls1 ≤ l2)* and *(d1 ≤ ls2 ≤ d2)*.

For each of the three patterns, one can either forbid insertions/deletions or allow them. In the former case, *Lsi = Li *and the weight = *W*i are computed as the sum of matching pairwise symbols, whereas in the latter case, the difference |*Lsi - Li*| between *spat(i) *and *pat(i) *is bounded by a value *Ri *for the permissible deletions/insertions (gaps), an optimum alignment, and its weight, *Wi*, are computed by the standard dynamic programming method for global string alignment [42]. In both cases, a symbol scoring matrix *Mi(x,j) *is used to define the weight of the symbol *x *in the position *j, 1 ≤ j ≤ Lsi*, of *spat(i)*. If symbol *x *occurs in position *j *of *pat(i)*, then *Mi(x,j) = 1*, otherwise *Mi(x,j) ≤ 1*. To choose the best approximation of the triad pattern from substrings satisfying conditions (i) and (ii), FIND_TRIAD uses a total score function with the form:

(1) tot_sc = C1*nsc1(L1,W1)+D12*nsc_dist12(11,l2,ls1) + C2*nsc2(L2,W2) + D23*nsc_dist23(d1,d2,ls2) + C3*nsc3(L3,W3),

where *nsci(Li,Wi), i = 1,2,3*, are normalized scores of matching (alignments) of *pat(i) *and *spat(i), 0 ≤ nsci(Li,Wi) ≤ 1*, and *nsc_dist12(11,l2,ls1) *and *nsc_dist23(d1,d2,ls2) *are the normalized scores of the distances between the first and the second, and the second and the third patterns, respectively, and *0 ≤ nsc_dist12(11,l2,ls1), nsc_dist23(d1d2,ls2) ≤ 1*. The linear coefficients *C1, C2, C3, D12*, and *D13 *are chosen so that their sum is equal to 1. They indicate the relative importance of the corresponding sub-patterns of the triads; and the distances between them. So, the best value of *tot_sc *is 1.

### Application of the algorithm to searching for strong promoter candidates

Here we describe the main parameters of the FIND_TRIAD procedure used to detect strong promoter candidates in bacterial genomes. In this study, a bacterial promoter is assumed to be a nucleotide sequence, located upstream from genes encoding proteins, tRNAs or rRNAs that could be recognized by an RNA polymerase holoenzyme containing a major σ factor (using *E. coli *Eσ^70 ^RNAP as the reference). The triad patterns defined for strong promoter candidates include three specific nucleotide sub-regions: (i), a UP element, which is a 17-nt prefix of the strong promoter, and has the following consensus pattern: *pat(1) *= ***P_UP _***= *aaaWWtWttttNNNaaa*; (ii) the -35 site, which is located downstream of the UP element, and has the pattern *pat(2) *= ***P_35 _***= *tcttgacat *(underlining indicates a commonly used consensus for group I σ^70 ^factors; however, the σ_4 _domain of these factors appears to be in contact with 9 nucleotides in the region extending from -30 to -38 [43,44]); (iii) the -10 site, which is located downstream of the -35 site, and has the pattern *pat(3) *= ***P_10 _***= *tataat *(this site is highly conserved). We used the following boundaries for the distances between the sub-regions: l1 = 0, l2 = 5 (these boundaries were extracted from the examples of UP-elements in [25-27]), d1 = 14, d2 = 20 (these boundaries are standard for the distance between the -35 site and the -10 site). To search for the first pattern *pat(1) *of the UP-element, the simple matching algorithm was chosen with an *a *and *t *mismatch score of 0.5. The reason is that in the full UP-element consensus and the consensuses of two of its subsides – distal and proximal – in some places do not distinguish between *a *and *t*. We assumed that the consensus for the -35 site of length 9 is less conserved than that of the -10 site, and so in order to detect the second pattern *pat(2*) of the -35 site we used a dynamic programming algorithm to search for optimal alignment, with boundaries for the number of permissible deletions/insertions of *R2 = 2*. For the most of -35 sites, which were detected by algorithm, no insertions/deletions were applied. However, this scoring system allowed us to identify some stronger promoter candidates. Thus, the insertion of C between two AA in the sequence TCTTGAAT of TM1016, increases the score of a putative promoter (see below). The -10 site is better conserved, and so we used the straightforward matching algorithm to detect this site.

To define the total score function, *tot_sc *(formula 1), we chose the following normalized scores for the three patterns and for the distance between the -35 site and -10 site (no information was available about the best values for the distance between the UP element and the -35 site):

(2) ***nsc1(17,W1) ***= *nsc_up = 1 - (17 - W1)/20*,

(3) ***nsc2(9,W2) ***= *nsc_35 = 1 - (9 - W2)/10*,

(4) ***nsc3(6,W3) ***= *nsc_10 = 1 - (6 - W3)*^2^*/10*,

and the values of the normalized distance score, *nsc_dist23(14,20,ls2) = nsc_dist*, are defined as follows:

$$\begin{array}{lllll} ls2:\text{distance between the -35 and -10 sites, nt} & 17 & 16,18 & 15,19 & 14,20\\ nsc\_dist & 1 & 0.95 & 0.85 & 0.7 \end{array}$$

We also chose linear coefficients *C1 = 0.3, C2 = C3 = 0.25, D12 = 0, and D23 = 0.2*. These coefficients indicate the relative importance of corresponding sub-regions for evaluating the total score of a candidate sequence. They were chosen empirically, after preliminary tests with several annotated genomes, assuming a higher significance of the UP element, equal significance of the -10 and -35 boxes, and lower significance of the distance between them. In this application, the value *D12 = 0 *means that we ignore the variations of the distance between a putative UP element and -35 box because *a priory *it is not known what value is the best in the interval 0–5 nt.

Formulas 2, 3 and 4 reflect the lack of exact matching for the different sub-regions. If the -10 box is highly conserved and is essential for initiation of transcription [22], then the penalty for its mismatches is higher than for those of the other parameters. For example, for 2 mismatches, the penalty is *(6 - 4)^2^/10 = 0.4 *for the -10 site, whereas it is *(9 - 7)/10 = 0.2 *for the -35 site, and *(17 - 15)/20 = 0.1 *for the UP element. The choice of the normalized score functions in equations 2, 3 and 4 is based on empirical observations, and on common sense, and may seem to be arbitrary. We want to stress that, in fact, the total score function *tot_sc *also has a further role: it does not significantly change the set of the best candidates identified by the algorithm. This set is defined by the three score bounds *Sc1 = scup *for UP element, *Sc2 = sc35 *for -35 site, and *Sc3 = sc10 *for -10 site. The total score affects only the ordering of these candidates amongst themselves.

The general scheme of the algorithm is as follows. It has the following input: (i) the name of a genome file in GenBank format; (ii) three parameters of scores: *scup, sc35 *and *sc10*, determining the minimum acceptable similarity between candidate sequences of the UP element, the -35 box, and the -10 box, respectively, and the *E. coli *consensus patterns. For each gene in the genome input file that is not inside an operon, the algorithm runs in two steps:

(i) it extracts a 300-bp DNA region, *s*, upstream of the annotated coding sequences for tRNA, rRNA or proteins (we limited the search to 300 bp, since most *E. coli *promoters fall within this length inter-gene space [41,45]);

(ii) then it uses the FIND_TRIAD procedure to identify the best strong promoter candidate within *s *that satisfies conditions (1) and (2) above. If such a candidate is found, it is added to the output list of strong promoters.

**Figure 1:**
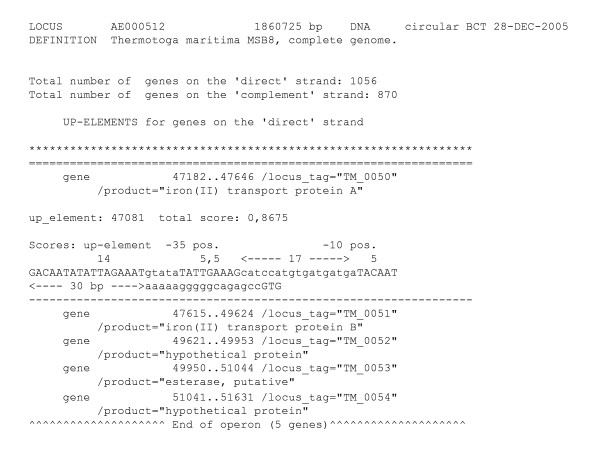
Text-format presentation of strong promoter candidates.

We recommend to read attentively the "ReadMe" information [see Additional file 1] before to start proceeding the "strong_promoters.doc" software [see Additional file 2]. The algorithm is implemented by a program that produces the results in two forms: (i) a Text-format table which lists all strong promoter candidates in a genome, and provides additional information about the operon organization of genes located downstream (for example, see Fig. 1); (ii) a Word-format table which lists strong promoter candidate sequences. A 20-nt sequence preceding a possible initiation codon of each ORF is also included in the annotation, as this could be useful for the visual examination of the translation signals of the corresponding genes. Lastly, the user can select a convenient score for each sequence-specific motif taking into consideration the promoter features of the annotated genome if they differ from the *E. coli*-specific patterns used to create the algorithm (for example, a weakly conserved -35 or -10 box).

## Methods

### Construction of recombinant linear DNAs

**Figure 2:**
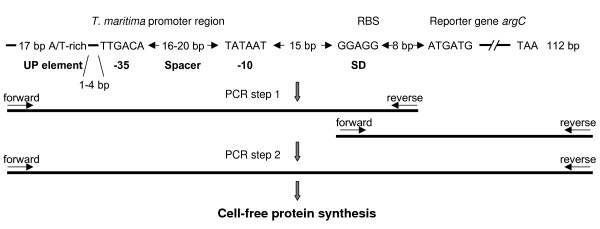
**Diagram of the fusion DNA constructs used to express the *G. stearothermophilus argC*-reporter gene from putative strong promoters of *T. maritima *in a cell-free system**. The *argC *gene was amplified with forward 5'-GGAGGGGGAACATATGATGAA and reverse 5'-GGACCACCGCGCTACTGCCG primers from pHAV2 [32] by conserving a 112-bp downstream region carrying transcriptional terminators of the vector DNA.

Putative promoter regions in the *T. maritima *genome, identified by the algorithm described above, were amplified by PCR using appropriate oligonucleotide primers connected to the previously-described *G. stearothermophilus argC *gene [46]. This reporter gene encodes *N*-acetyl glutamylphosphate reductase, a thermostable and soluble protein that is easily detectable after exposing *E. coli *cleared lysates to 65°C. In order to increase protein yield, the ribosome-binding site of *G. stearothermophilus argC *was modified to the sequence GGAGGGGGAACATATG (the modified Shine-Dalgarno site and the initiation codon are underlined), and the distance between the -10 promoter site and the Shine-Dalgarno site was shortened to 15 bp (Fig. 2). The DNA fragment carrying the *argC *gene was connected to *T. maritima *or control promoters by two consecutive PCR steps, as described previously [47]. The quantity and quality of the amplified DNAs were determined with a 2100 Bioanalyzer (Agilent Technologies).

Two well-characterized strong promoters, P*tac *and P*argC*, were used as references to compare the strength of the putative promoters of *T. maritima*. The strong promoter P*tac *contains an AT-rich nucleotide sequence upstream of a -35 site [48], which has no defined UP element; it was obtained from the vector pBTac2 (purchased from Boehringer Mannheim). P*argC*, a strong promoter of *G. stearothermophilus*, contains the UP element, as demonstrated both *in vivo *and *in vitro*, and was amplified from the plasmid pHAV2 [32].

### Cell-free protein synthesis

PCR-generated linear DNA fragments carrying a promoter region fused to the *argC *reporter gene were used to evaluate the promoter strength in a coupled transcription-translation system, as described previously [49]. The cell-free extracts were prepared from the *E. coli *strain BL21 (DE3) Star *recBCD *(our laboratory construction) as described by Pratt [50]. Protein synthesis was carried in the presence of pyruvate oxidase to generate ATP [51]. Typically, 50 ng of PCR-amplified DNA was added to a pre-mix containing all necessary compounds and 10 μCi of [α^35^S]-L-methionine (specific activity 1000 Ci/mmol, 37 TBq/mmol, Amersham-Pharmacia Biotech), and *E. coli *S30 cell-free extracts. The reaction mixture was incubated at 37°C for 90 min, and heated to 65°C for 10 min. After centrifuging, the supernatant was precipitated with acetone, and then protein samples were separated by SDS-PAGE and bound to 3 MM paper. The ArgC protein synthesized *in vitro *was quantified by counting the radioactivity of the corresponding band with a PhosphorImager 445 SI (Molecular Dynamics).

The bacterial genome sequences were extracted from available data banks. The logo of *T. maritima *promoter consensus sequences was generated at the WebLogo site as described in [52,53].

## Results

### The number of strong promoters reflects the A+T content of bacterial genomes

In our algorithm, 26 of the 32 symbols used to evaluate matches in the three promoter-specific patterns, namely in the UP element and the -35 and -10 boxes, are *a *and *t*. One could expect the number of genes transcribed from potential strong promoters to depend on the A+T content of a given genome. To find out whether this is indeed the case, we compared the frequency of candidates in 300-bp regions located upstream of genes of annotated bacterial genomes and in random sequences of the same regions generated by computing. First, we calculated the (A+T)% in all 300-bp regions preceding each gene or operon in the annotated genomes (Table 1). The A+T content in these DNA regions was found to be slightly higher than that of the entire genomes of almost all bacteria that have been analyzed. Next, we generated 10.000 random sequences with the same A+T content for all the 300-bp regions of each genome. The algorithm was applied to detect strong promoter candidates in the 300-bp real genomic and random-generated regions of 43 bacterial genomes.

**Table 1:** A+T content of bacterial genomes and 300-bp regions located upstream of genes and the percentage of strong promoter candidates predicted in 300-bp real genomic and random-generated regions of the same content.

		(A+T)% of		% of candidates in	
			
N°	Genome	Bacterial genomes*	300-bp genomic regions	300-bp genomic regions	300-bp random sequences
1	*Deinococcus radiodurans *R1 (AE000513)	32.99	34.19	0.19	0
2	*Caulobacter crescentus *(AE005673)	32.77	34.40	0.05	0
3	*Ralstonia solanacearum *GMI1000 (AL646052)	34.51	34.50	0.20	0
4	*Pseudomonas aeruginosa *PA01 (AE004091)	33.44	35.38	0.27	0
5	*Xanthomonas campestris pv. campestris *(AE008922)	34.93	35.64	0.05	0
6	*Mycobacterium tuberculosis *(AL123456)	34.39	35.69	0.18	0
7	*Xanthomonas axonopodis pv. citri *306 (AE008923)	35.23	36.02	0.05	0
8	*Mesorhizobium loti *(NC002678)	37.25	39.09	0.13	0
9	*Sinorhizobium meliloti *1021 (AL591688)	37.27	39.66	0.24	0
10	*Mycobacterium leprae *TN (AL450380)	42.20	43.14	0.29	0
11	*Agrobacterium tumefaciens *C58 (AE007869)	45.64	43.20	0.74	0
12	*Brucella melitensis *16 M chromosome I (AE008917)	42.84	45.73	1.02	0
13	*Treponema pallidum *(AE000520)	47.22	47.01	0.37	0.3
14	*Chlorobium tepidum *TLS (AE006470)	43.47	47.50	1.50	0.31
15	*Salmonella typhimurium *LT2 (AE006468)	47.78	51.08	3.54	0.7
16	*Neisseria meningitidis *serogroup B MC58 (AE002098)	48.47	52.20	5.03	0.93
17	*Escherichia coli *O157:H7 (AE005174)**	49.50	52.54	4.80	1.1
18	*Methanobacterium thermoautotrophicum *ΔH (AE000666)	50.46	53.11	4.26	1.23
19	*Synechocystis *PCC6803 (AB001339)	52.28	53.71	2.89	1.7
20	*Thermotoga maritima *(AE000512)	53.75	54.66	3.27	2.15
21	*Vibrio cholerae *chromosome I (AE003852)	52.30	54.94	3.22	2.4
22	*Yersinia pestis *CO92 (AL590842)	52.36	55.77	6.78	3.0
23	*Aquifex aeolicus *(AE000657)	57.73	57.70	4.72	4.15
24	*Bacillus halodurans *C-125 (BA000004)	56.31	58.65	8.70	5.1
25	*Bacillus subtilis *(AL009126)	56.48	59.30	10.28	5.8
26	*Mycoplasma pneumoniae *M129 (U00089)	59.99	61.71	5.25	9.7
27	*Chlamydia muridarum *(AE002160)	59.69	61.73	9.01	9.7
28	*Pasteurella multocida *PM70 (AE004439)	59.60	62.31	11.42	10.9
29	*Chlamydophila pneumoniae *J138 (BA000008)	59.42	62.80	14.77	12.5
30	*Streptococcus pneumoniae *(AE005672)	60.30	62.88	15.83	13.0
31	*Streptococcus pyogenes *SF370 serotype M1 (AE004092)	61.49	63.99	16.87	14.3
32	*Thermoanaerobacter tengcongensis *MB4T (AE008691)	62.43	64.11	17.74	14.8
33	*Listeria innocua *Clip11262 (AL592022)	62.56	64.30	12.07	15.5
34	*Haemophilus influenzae *Rd (L42023)	61.85	64.45	15.61	16.0
35	*Mycoplasma genitalium *G37 (L43967)	68.31	69.50	15.99	35.0
36	*Staphylococcus aureus *N315 (BA000018)	67.16	69.71	35.25	36.1
37	*Campylobacter jejuni *(AL111168)	69.45	71.36	32.07	41.8
38	*Clostridium acetobutylicum *(AE001437)	69.07	71.83	45.08	44.2
39	*Borrelia burgdorferi*.(AE000783)	71.40	73.18	40.00	54.1
40	*Rickettsia prowazekii *Madrid E (AJ235269)	71.00	73.26	50.06	55.2
41	*Clostridium perfringens *13 (BA000016)	71.43	74.74	53.94	58.1
42	*Ureaplasma urealyticum *(AF222894)	74.50	76.05	50.85	65.35
43	*Buchnera aphidicola Sg *(AE013218)	74.67	78.36	58.05	74.5

* A+T content of bacterial genomes was calculated from corresponding genomic DNA sequences available in gene banks.

** Similar values were found for the *E. coli *K12 genome.

We tested different matching stringencies and empirically found that the score parameters *sUP *= 13, *s35 *= 5.5 and *s10 *= 4.5 satisfied the criteria required for scaled comparative analysis without grossly exaggerating the number of candidate sequences identified in the various genomes. This analysis revealed that the real genomes with an A+T content of less than 50% contained many more potential strong promoters than their simulated counterparts (see Table 1). The percentage of candidate sequences was very low in the bacterial genomes with an A+T content of between 33% and 47%, and these sequences were completely absent in the corresponding 300-bp, random-generated sequences. When the A+T content increased from 47% to 78%, the percentage of strong promoter candidates increased dramatically, whereas the difference between the real and random sequences decreased, and virtually disappeared when the A+T content exceeded 62%. There were two exceptions where the genomes analyzed did not display this pattern at an A+T content of less than 62%. One was *M. pneumoniae*, the genome of which had an A+T content of about 60%, and in which the promoters had no -35 consensus [54]. The other example is the hyperthermophilic species *A. aeolicus *(~58% AT-rich genome). This species is very close to the Archaea, and occupies a unique position in the bacterial kingdom [55].

**Figure 3:**
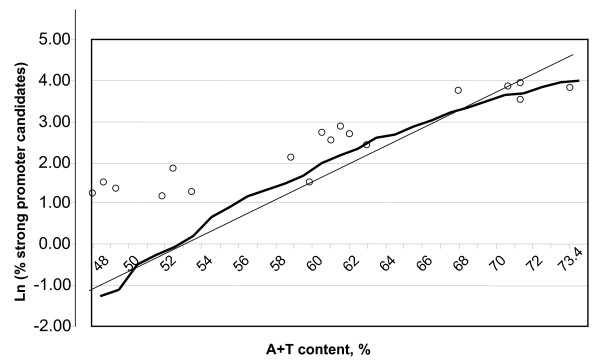
**The number of strong promoter candidate sequences is a function of the A+T content of bacterial genomes**. For the score parameters *sUp *= 13, *s35 *= 5.5, *s10 *= 4.5 and constants c_1 _= 0.22 and c_2 _= -11.7, the picture displays a linear graph of the "exponential low" (thin line), which approximates fairly closely to the curve ln [N(A+T)], shown as a thick line. The logarithm of the percentage of strong promoter candidates in real genomes is shown by (○).

Our data show that the number N(A+T) of strong promoter candidates in 300-bp random-generated sequences corresponding to upstream regions of bacterial genes satisfies the "exponential low" of the form N(A+T) = exp [c_1 _(A+T) + c_2_]. The distribution of strong promoter candidates in real genomes indicates that the critical point of the A+T content is close to 62% (Fig. 3). Above this level, the number of random sequences reminiscent of strong promoter patterns increases markedly.

### Strong promoter candidate sequences are located upstream of gene-coding regions

Another important aspect of the quality of detection is the location of candidate sequences with regard to coding regions in the genome analyzed. We compared the frequencies of strong promoter-like patterns identified upstream and downstream of the initiation codon in all the genomes. The frequency of candidate sequences was clearly greater in the upstream region of ORFs in most of the genomes with an A+T content of less than 62% (Table 2). No difference was detected in *T. pallidum *(~47% AT-rich genome), which belongs to a distinct phylum of Spirochetes that appear to use different DNA patterns for the promotion and regulation of transcription [56].

**Table 2:** Number of sequences reminiscent of strong promoters in regions located upstream and downstream of the initiation codon of genes in bacterial genomes.

N°	Genome	Length, bp	Number of genes	Upstream region	Downstream region
1	*Deinococcus radiodurans *R1 (AE000513)	2648638	2681	5	1
2	*Caulobacter crescentus *(AE005673)	4016947	3787	2	0
3	*Ralstonia solanacearum *GMI1000 (AL646052)	3716413	3477	7	0
4	*Pseudomonas aeruginosa *PA01 (AE004091)	6264403	5570	15	2
5	*Xanthomonas campestris pv. campestris *(AE008922)	5076188	4197	2	0
6	*Mycobacterium tuberculosis *(AL123456)	4411529	3922	7	0
7	*Xanthomonas axonopodis pv. citri *306 (AE008923)	5175554	4344	2	0
8	*Mesorhizobium loti *(NC 002678)	7036074	6693	9	0
9	*Sinorhizobium meliloti *1021 (AL591688)	3654135	3375	8	0
10	*Mycobacterium leprae *TN (AL450380)	3268203	2770	8	1
11	*Agrobacterium tumefaciens *C58 (AE007869)	2841581	2701	20	1
12	*Brucella melitensis *16 M chromosome I (AE008917)	2117144	2059	21	4
13	*Treponema pallidum *(AE000520)	1138011	1083	4	3
14	*Chlorobium tepidum *TLS (AE006470)	2154946	2329	35	13
15	*Salmonella typhimurium *LT2 (AE006468)	4857432	4608	163	61
16	*Neisseria meningitidis serogroup B *MC58 (AE002098)	2272351	2226	112	45
17	*Escherichia coli *O157:H7 (AE005174)	5528445	5478	263	79
18	*Methanobacterium thermoautotrophicum *delta H (AE000666)	1751377	1900	81	24
19	*Synechocystis *PCC6803 (AB001339)	3573470	1074	31	6
20	*Thermotoga maritima *(AE000512)	1860725	1926	63	10
21	*Vibrio cholerae *chromosome I (AE003852)	2961149	2887	93	37
22	*Yersinia pestis *CO92 (AL590842)	4653728	4042	274	61
23	*Aquifex aeolicus *(AE000657)	1551335	1503	71	37
24	*Bacillus halodurans *C-125 (BA000004)	4202353	4125	359	87
25	*Bacillus subtilis *(AL009126)	4214814	4182	430	111
26	*Mycoplasma pneumoniae *M129 (U00089)	816394	705	37	14
27	*Chlamydia muridarum *(AE002160)	1069411	954	86	31
28	*Pasteurella multocida *PM70 (AE004439)	2257487	1996	228	64
29	*Chlamydophila pneumoniae *J138 (BA000008)	1226565	1097	162	51
30	*Streptococcus pneumoniae *(AE005672)	2160837	2306	365	156
31	*Streptococcus pyogenes *SF370 serotype M1 (AE004092)	1852441	1731	292	115
32	*Thermoanaerobacter tengcongensis *MB4T (AE008691)	2689445	2632	467	248
33	*Listeria innocua *Clip11262 (AL592022)	3011208	3529	426	229
34	*Haemophilus influenzae *Rd (L42023)	1830138	1775	277	94
35	*Mycoplasma genitalium *G37 (L43967)	580074	519	83	63
36	*Staphylococcus aureus *N315 (BA000018)	2814816	2638	930	418
37	*Campylobacter jejuni *(AL111168)	1641481	1684	540	353
38	*Clostridium acetobutylicum *(AE001473)	3940880	3738	1685	916
39	*Borrelia burgdorferi *(AE000783)	910724	875	350	292
40	*Rickettsia prowazekii *Madrid E (AJ235269)	1111523	885	443	252
41	*Clostridium perfringens *13 (BA000016)	3031430	2779	1499	772
42	*Ureaplasma urealyticum *(AF222894)	751719	645	328	236
43	*Buchnera aphidicola Sg *(AE013218)	641454	584	339	225

The fact that more candidate sequences were identified upstream of ORFs highlights the fact that they are not randomly distributed in bacterial genomes, which suggests that the detection of strong promoter candidates in genomes with an A+T content of less than 62% is fairly reliable.

### Experimental validation of virtual prediction: analysis of putative strong promoters of *T. maritima*

Taking our cue from the results of the virtual prediction, we sought to find out whether, and if so, to what extent the putative promoters are functional in a biological context. To do this we used reporter-gene technology, which relies on the fusion of an assayable sequence with a promoter being investigated, and the subsequent evaluation of promoter strength in a cell-free system (see Fig. 2). The genome of the hyperthermophilic bacterium *T. maritima *[57] was used to evaluate the feasibility of the algorithm experimentally.

63 candidate sequences were detected in the *T. maritima *genome using the matching scores described above. We increased the penalty for mismatching of -35 and -10 boxes by raising the scores of *s35 *and *s10 *to 6 and 5, respectively. This reduced the number of candidate sequences to 34 (Table 3). In this shorter list, 28 *T. maritima *strong promoter candidates possessed a total score higher than the 0.8475 calculated for the reference strong promoter, P*tac*, that does not have a typical UP element [48]. 15 of these candidates had a total score higher than 0.8775, as estimated for P*argC*, another reference strong promoter that has a well defined UP element [32,49]. It is worth mentioning that 6 candidate DNA regions in *T. maritima *had a total score higher than 0.91, a value estimated for *E. coli *promoters that govern the transcription of 16S ribosomal RNA, and which were used as models for studying the stimulating effect of the UP element on gene expression [58].

**Table 3:** Strong promoter candidates identified in *T. maritima *MSB8*.

Downstream located gene(s)**	Strong promoter candidate sequence***	Total score****
TM_0013 conserved hypothetical protein Operon: 2 genes	ACAATTTTTATCTGATATTTTTTTCACAttcaccatagtcgatTATAAC <--- 97 bp --->aatctggaggtgacaatATG	0,8475
TM_0110 transcriptional regulator, XylR-related Operon: 6 genes	ACCTTGATTTTAAATTATTTCCTGCATataattaatgtgaaCATAAT <--- 10 bp --->aaaaggaggaatcgaagTTG	0,805
TM_0280 hypothetical protein Operon: 6 genes	GCAATATTTGTCCAGAAATATACTTGATTtaacaaaaatggacaatgTAGAAT <--- 37 bp --->aaggaggaatatcgtttATG	0,88
TM_0339 hypothetical protein Operon: 3 genes	AGAAAAATTTTTTTGGAGACTTGACAaaatatttggtaatattcTAAAAT <--- 5 bp --->gcaggaggtgacaaaatATG	0,8975
TM_0373 dnaK protein Operon: 2 genes	TTTTACAAATTCTCATACGACCCCTTGACAtcccattctgtgcctcacTATAAT <--- 21 bp --->tctaaggaggtgacacaATG	0,94
TM_0657 rubrerythrin Operon: 3 genes	TAATGTAACTATTCAAAATCATTACAgtttataattatgtggTAAAAT <--- 22 bp --->atagggaggtgcagggtATG	0,8125
TM_0682 hypothetical protein Operon: 3 genes	GAATACTCTGTCAGAAAGATTCGTGATCAtcttttcacctcgtgtagTATAAT <--- 7 bp --->gagtattcttctacacaATG	0,915
TM_1016 hypothetical protein	TAAAAATTTCATGAAAAATTTCTTGAATtctgtgaccaaaagggTTTAAT <--- 5 bp --->gccggaggtgatgtgagATG	0,9175
TM_1167 hypothetical protein	GAAAAGTTACAGAAAAAGTACCCTTGTTAtctgaaggtgaaaaatggTAAAAT <--- 61 bp --->tacagggagggcgggagATG	0,865
TM_t27 tRNA-Asn	TCATTCATTTTACCATCGAGTCCACTTGAAAttcaggaaggtatgtagTACAAT <--- 0 bp --->tatccgtggaggttcc	0,8675
TM_1205 conserved hypothetical protein Operon: 13 genes	GTTTTTTATCTCTACTAATTAGGTTGACAttattgattcagaagagTAAAAT <--- 40 bp --->ccgaggaggtgtgatgaGTG	0,88
TM_1318 Operon: 2 genes	AGAAACAATTTTGGAATTTGATCCATGGACAttattacctttaatgGATAAT <--- 0 bp --->tttaacggaggATG	0,8325
TM_t34 tRNA-Leu	AGAAAAATTTCCGATGAGGGTACTTGAAAagggtgaaaacctgtgcTATTAT <--- 0 bp --->atatgtcggagttgcc	0,855
TM_1429 glycerol uptake facilitator protein Operon: 3 genes	GCATTGTGATTTTTGTAACTATATTGACAtaaaacaaaaggtttgtTATAAT <--- 107 bp --->caaggaggattgggaaaATG	0,9175
TM_t39 tRNA-Ala Operon: 3 genes	AAAAATAAAAAGTCCTTCTGGGGATTGACCatatttcgtactcatgcTATAAT <--- 50 bp --->taatataaagacgaggtggg	0,8725
TM_1667 xylose isomerase Operon: 2 genes	AAGTATATCCTAAAAAAATATTTGAAAtgataccccaagattttaTATAAT <--- 16 bp --->tttagggaggtgtttacATG	0,905
TM_1780 argininosuccinate synthase Operon: 6 genes	GAAAATAACAGTGAAAAAACACTTCATAtaaatcatttcaaataatccTATAAT <--- 15 bp --->aaagaggagggttcatcATG	0,875
TM_0150 (complem.) ribosomal protein L32 Operon: 5 genes	AAAAATGTAAAAGAAGAGAAACTTGAATctttgaaaaacatcaTATACT <--- 210 bp --->acgaggaggtataaaagATG	0,855
TM_0477 (complem.) outer membrane protein alpha	ACAAAAAAACTTTAGAAAACTCTTGAATttcctttggacgggatggTATAAT <--- 28 bp --->gaagggaggtttgtcccATG	0,9425
TM_0625 (complem.) hypothetical protein	ATATTCGTTCTGAATGAAGGTTTTACATttcatccaaattattttggtTATAGT <--- 152 bp --->attggaggcaaatagaaATG	0,805
TM_0656 (complem.) conserved hypothetical protein Operon: 2 genes	AACTTAAGTAACACAAAATTAACCTTGACAacgaaaggggggtgggTATAAT <--- 42 bp --->aaggggttgggaactttGTG	0,8925
TM_0755 (complem.) conserved hypothetical protein Operon: 2 genes	AGAAATTCTTTGAAAACTATCTAGAATtcaaacgtcgcttttccagTATACT <--- 101 bp --->aatggaggtgtctctgtATG	0,85
TM_0971 (complem.) hypothetical protein	AAATATAAATCTGAATTTACTAAATTCACAtttagcaaatcatcattTATAAT <--- 10 bp --->aggaatctcaagggggaATG	0,895
TM_1015 (complem.) glutamate dehydrogenase	ATAATTTTTGCAATTTTATCTCTATACAtctcacatcacctccggctaTATATT <--- 104 bp --->ttcgaggggggaaatgtATG	0,855
TM_1067 (complem.) oligopeptide ABC transporter, periplasmic	GGATTATTTTATACTGAAAGCCCTTGACCttgttgtatgtttgttgaTATTAT <--- 45 bp --->ataacgcagggggtggtATG	0,92
TM_1271 (complem.) type IV pilin-related protein	GGGTGATATTTCAACATTAAAATCTTGACAttctaccatgtcaaggtgTATAAT <--- 35 bp --->cccgggaggtggattttATG	0,9525
TM_1286 (complem.) 5-methyltetrahydropteroyltriglutamate...	GTTTATGCAAATTTTCCTTCTGTTAACCAtgttacacacaacatgtggTATCAT <--- 19 bp --->aatggaggtgaaaagggTTG	0,8625
TM_t31 (complem.) tRNA-Leu	AAGTTTTGATTTTTGTAAGGTTGAAAtaatctttctgacgatgtggTATAAT <--- 0 bp --->aaaaaaaggagcc	0,86
TM_1412 (complem.) hypothetical protein	ATATGGAAGTTCAAAAAACATCTTGCTTtcagagtgtgtttgtggTATAAA <--- 24 bp --->aataattccttagaggtATG	0,865
TM_1419 (complem.) myo-inositol-1-phosphate synthase-... Operon: 3 genes	AGAAAACTATTGGTAAAGCACTTGAAAtatatgactgtaaaaacgtgaTATAAT <--- 61 bp --->ctaaggaggtgaaacatATG	0,87
TM_1439 (complem.) hypothetical protein Operon: 3 genes	TAGTATTCTACCCTAAACTCTTTCAttctggattcgataatTGTAAT <--- 222 bp --->tgagagtgaaaaaggccATG	0,835
TM_t45 (complem.) tRNA-Ser	AAAAGAAGGAAGAAAAATGAAAACTTGAACaaggaaacgattgagtgTATAAT <--- 1 bp --->tttttctggtgtggagagga	0,865
TM_1786 (complem.) hypothetical protein	GTATTATTCATTCTAAAAACTTGAAActgaccaaataaagtatTAGAAT <--- 44 bp --->cacaagggggtgttttcATG	0,855
TM_1850 (complem.) hypothetical protein	AAACGATTCTTCTAAAATGTGTTCTTGATTtgtatcactgttatgtTATAAA <--- 43 bp --->aaaaaggaggtgaaactATG	0,855
TM_t11 tRNA-Thr Operon: 2 genes	GAAAAGGGTTATCAGGAAATATCTTGAATagaaaaggttcgtgtgtTAAAAT <--- 0 bp --->aaccacagaggcgagca	0,8825
TM_1272 glutamyl tRNA-Gln amidotransferase... Operon: 3 genes	TTTCACATTTTGCATTATACACCTTGACAtggtagaatgtcaagatTTTAAT <--- 99 bp --->ataatccacgagaggagGTG	0,8975
TM_0032 (complem.) transcriptional regulator, XylR-related Operon: 1 genes	AATATTAGAATTTGAACTATAATTCGAAAtaattcctgttattcactCATAAT <--- 79 bp --->agcaggaggaatatggaGTG	0,86
TM_1490 (complem.) ribosomal protein L14 Operon: 22 genes	GGTGAAAATATGCCCAGGAAACGTTTGACTggaatagttgtgagcgaTAAAAT <--- 259 bp --->aagggaggggttgaatcATG	0,845

* The genome annotation of *T. maritima *AE000512 used for analysis was dated 28^th ^December 2005.

** The gene order for the first 34 candidate sequences is shown on both strands as described in the annotated genome [49]. The complementary strand is noted as (complem).

*** The spacer between -35 and -10 sites and the region located downstream of the -10 site are shown in lowercase; the initiation codons of the ORFs are shown in capital letters at the end of the corresponding sequences.

**** The first 34 candidate sequences were detected with the score parameters *sUP *= 13, *s35 *= 6, *s10 *= 5; TMt11, TM1272, TM0032 and TM1490 were detected with *sUP *= 12, *s35 *= 6, *s10 *= 5 and used for analysis in a cell-free system (see Fig. 3).

**Figure 4:**
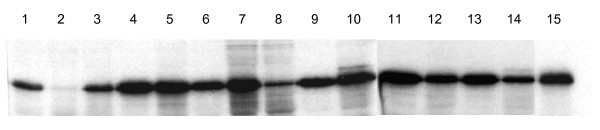
**Assessment of the strength of *T. maritima *strong promoter candidates in a cell-free system**. Lanes 1 – P*tac *(reference); 2 – P*TM0032*; 3 – P*TM0373*; 4 – P*TM0477*; 5 – P*TM1016*; 6 – P*TM1067*; 7 – P*TM1271*; 8 – P*TM1272*; 9 – P*TM1429*; 10 – P*TM1490*; 11 – P*TM1667*; 12 – P*TM1780*; 13 – P*TMt45*; 14 – P*TMt11*; 15 – P*argC*. Similar results were obtained in 3 experiments.

We selected 13 candidate promoter sequences for further analysis by evaluation of the ArgC thermostable protein production in a coupled transcription-translation system. These sequences all exhibited a total score ≥ 0.8475, apart from TM1490 (see Table 3). The amplified DNA regions were connected to the reporter gene *argC*, and used directly to assess promoter activity *in vitro *(see Fig. 2). All putative promoters of *T. maritima *were found to be active; the protein yield ranged from 0.3 to 2.7-times that of the reference P*tac *promoter (Fig. 4). The gene expression from the promoter P*TM1272 *was similar to that of P*tac*, whereas P*TM0032 *was reduced almost threefold. However, higher expression was detected from the other 11 promoters; the greatest expression level was observed for P*TM0477*, P*TM1016*, P*TM1429 *and P*TMt45*. Reporter gene expression was also higher for the strong promoter P*argC*, which carries the UP element.

**Figure 5:**
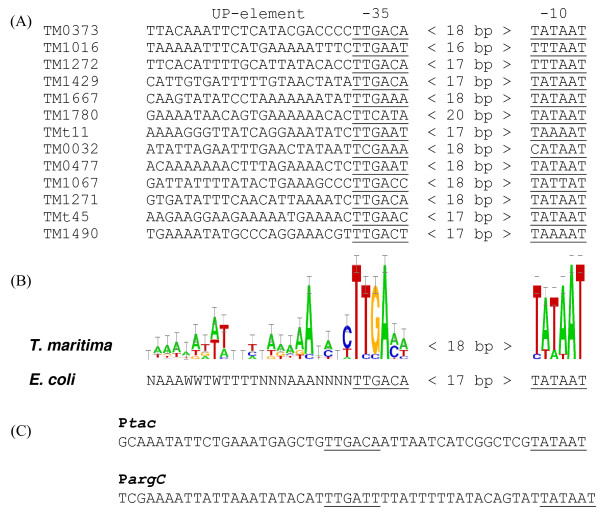
**Organization of strong bacterial promoters**. (A), Alignment of 13 promoter candidates of *T. maritima*; (B) consensus sequences of *T. maritima *and *E. coli *strong promoters; consensus of the *E. coli *UP element is described in [26, 27]; (C) the strong promoters P*tac *and P*argC *were used as references in this study.

We next aligned experimentally analyzed promoters of *T. maritima *(Fig. 5). The most conserved sequence was the -10 box, which was identical to the *E. coli *consensus. The -35 box was also highly conserved, except that cytosine preceded the -35 site in 9 promoters, and no significant preference was detected for the nucleotides at the 5^th ^and 6^th ^positions. An 18-bp spacer appeared to be more representative than a 17-bp distance between the -35 and -10 boxes. Although all candidates possessed an AT-rich region upstream of the -35 site, some of them had only one A-rich tract, suggesting that they harbor only a single sub-site of a putative UP element. In any case, the high score attributed to 11 identified promoters was corroborated by elevated activity *in vitro*. Taken together, the alignment data and the expression data from the cell-free system, suggest that *E. coli *RNA polymerase efficiently recognizes putative strong promoters of *T. maritima*, and that the presence of an UP-like element might contribute to the strength of the promoter.

Two regions, (2.4 and 4.2) of the four domains of σ^70 ^are involved in the recognition of the -10 and -35 boxes of *E. coli *promoters, respectively [59]. Several amino acids involved in contact with DNA have been also identified in the α subunit [60]. These DNA-binding regions in both σ^70 ^and α subunits of *E. coli *and *T. maritima *RNA polymerases share high similarity (data not shown), which highlights the fact that -35 and -10 boxes and UP-like element all contribute to the high promoter activity in the thermophilic host.

## Discussion

Bacterial promoters can be arbitrarily classified as weak, moderate and strong promoters, depending on the level of expression of mRNAs or of the corresponding proteins. We have developed an algorithm that can predict strong promoters in bacterial genomes by matching the triad pattern specific for the group I σ^70 ^factor of *E. coli *RNA polymerase. The first step in the proposed triad pattern approach involves matching the UP element located 300 bp upstream of a gene-coding sequence, and then matching two optimally separated -35 and -10 boxes.

The accuracy of the computational prediction of bacterial promoters depends on the A+T content of the genomes, which means that the matrix has to be adjusted to account for this factor in the DNA under analysis [29]. The data presented highlight the fact that the detection accuracy is lower in genomes with a high A+T content. The number of potential strong promoters identified in 43 bacterial genomes, is a direct function of their A+T content; this implies that the accuracy of the prediction is lower for genomes with A+T content higher than 62%.

The choice of the matching score is yet another difficulty in identifying DNA-binding sites including promoters, as the highest score may not be the one most biologically relevant for genome-scale predictions [61,62]. It is therefore helpful to use additional criteria to eliminate false-positives. It looks as if the total score of 0.8475, calculated for the reference promoter P*tac*, can be used as an reasonable criterion for identifying real strong promoters recognized by an Eσ^70^-like RNA polymerase. In particular, using the scores applied to genomes analysis (see Tables 1 and 2), the algorithm detects 7 potential strong promoters in *M. tuberculosis *(~34% AT-rich genome) that encodes a variety of σ factors, including σ^A ^that recognizes the promoters possessing typical -10 and -35 boxes [63]. However, none of the predicted strong promoters had a total score in excess of 0.8475, and visual inspection indicated that none of these promoters possesses an UP-like sequence, suggesting that this gene expression-stimulating element is absent in *M. tuberculosis*.

The possibility of applying linear PCR-generated molecules for cell-free protein synthesis, without needing to perform DNA cloning in bacteria, is a prerequisite for assessing gene expression on a genome-wide scale. As a first step in this direction, we tested reporter-gene fusions to evaluate the strength of the promoters identified in the genome of *T. maritima*. Though this approach does not exclude possible masking effects of *E. coli *repressors or activators in the extracts, it is relatively simple, timesaving and informative, all of which are major advantages for evaluating computational predictions. Using the two well-characterized strong promoters (P*tac *and P*argC*) as references, high activity has been demonstrated for 11 out of 13 candidate sequences of *T. maritima*. This is quite a low proportion; however, it suggests that the detection accuracy by the triad pattern algorithm might be close to 85%. The limitations of the algorithm in terms of specificity and sensitivity of the virtual prediction of putative strong promoters might be further experimentally evaluated by analysis of bacterial genomes with high-throughput methods.

This study offers the first insight into the organization and distribution of strong promoters in hyperthermophilic organisms, which probably constitute the longest lineage in the microbial world [64]. Overall, strong promoters of hyperthermophiles are similar to those of mesophilic origin. We have recently shown that the *T. maritima *RNA polymerase α subunit binds to the P*argG *promoter described here under P*TM1780 *[65]. It has been found that the substitution of arginine in the hyperthermophilic α subunit, corresponding to the position Arg265 in the *E. coli *subunit and crucial for DNA recognition [60,66], or the deletion of an AT-rich sequence located upstream of the -35 site, decreases the binding affinity for DNA [65]. The P*argG *promoter harbors a UP-like element, and is able to direct high gene expression *in vitro*. Moreover, this element appears to compensate for a poor -35 box or non-optimal 20-bp spacer of this promoter (see Table 3 and Fig. 5). Hence, these observations, along with the data obtained using other *T. maritima *promoters, allow us to assume that the presence of a UP-like element with less than 5 mismatches out of 17 nucleotides is essential for the strength of most strong promoters. This is consistent with the conservation of DNA interaction amino acids in the α subunit of the hyperthermoiphilic RNA polymerase. However, sequence-independent upstream DNA interactions within the C-terminal domain of the α subunit could often be required to initiate transcription in *E. coli *cells [67]. Therefore, the functional significance of the UP-like element in gene expression remains to be proven experimentally in hyperthermophilic organisms.

The strong promoters of *T. maritima *direct the transcription of genes involved in tRNA, ribosome synthesis, energy metabolism, transport, and cell movement (see Table 3). However, to our surprise, we found that 15 of the 38 best candidates promote the transcription of hypothetical proteins. The previously uncharacterized hypothetical protein TM1016 (total score 0.9175) turns out to share 28% identity with a biopolymer transport protein of *Vibrio vulnificus *[68]. In this context, recent studies of the *T. maritima *transcriptome have indicated that ABC transporters could play a major role in its ecology [69]. Further characterization of highly expressed hypothetical genes identified in our study might help to elucidate their role in the biology of this hyperthermophilic organism.

The strong promoter candidates prediction could contribute to the wide-scale genome expression analysis of evolutionarily distant bacteria, especially of those that possess an A+T DNA content lower than 62%. As a complement to DNA microarrays, it could help to elucidate the overall response of bacterial genomes to various environmental stresses. Moreover, the triad pattern algorithm can be used to extract the DNA region that carries translational signals; this is useful for investigating ORFs located downstream from the corresponding strong promoters (see Table 3). Thus, almost half of the *T. maritima *ORFs transcribed from putative strong promoters are preceded by a highly conserved Shine-Dalgarno site located 7–9 nucleotides from the ATG initiation codon, which is a characteristic feature of elevated protein synthesis in gram-negative and gram-positive bacteria [70]. This information will be useful for comparing highly synthesized mRNAs with the production of the corresponding proteins using high-throughput transcriptomic and proteomic methods, which is an important challenge in the fields of basic and applied microbiology [71]. Furthermore, the characterization of proteins whose expression is governed by strong promoters looks like a promising approach to selecting candidate vaccines against microbial diseases and/or to identifying potential new antibacterial targets in the fight against nosocomial infections.

Further quantitative assessment of a dynamic and complicated mechanism of protein-DNA and protein-protein interactions involved in transcription might help to develop a more advantageous multi-pattern tool using both DNA and protein parameters to provide a comprehensive prediction of the strength of promoter activity in bacterial cells.

## Conclusion

The triad pattern algorithm developed predicts strong promoter candidates by matching UP-like elements and identifying the presence of -35 and -10 boxes optimally distanced from each other in the annotated bacterial genomes. The presence of strong promoters is a function of the A+T content of the bacterial genome, and the number of false-positives is greater for genomes that have an A+T content higher than 62%. The prediction algorithm has been validated by cell-free experimental dissection of putative *T. maritima *promoters. The data indicate that strong promoters govern the transcription of genes coding vital functions, and of genes coding as-yet unknown functions in this hyperthermophilic bacterium. This algorithm is simple to use and flexible, and it could be further adapted to meet the requirements of a genome of interest if its promoter-specific motifs differ from consensi recognized by Eσ^70^-like RNA polymerase.

## Availability and requirements

The algorithm is freely accessible for non-commercial use at the web-site . It takes several seconds to analyze the annotated genome sequence available from databases.

## Competing interests

The authors declare that they have no competing interests.

## Authors' contributions

MD developed the algorithm and performed the computational analysis; AM conducted cell-free experiments; VS designed the project, contributed to the development of the algorithm and data analysis, and wrote the manuscript.

## Supplementary Material

Additional file 1 ReadMe. Contains information to use the algorithm. Click here for file

Additional file 2 Software "strong_promoters.doc". The Text-format provides the list of putative strong promoter sequences with total and individual scores obtained for each consensus. The Word-format provides the tabulated list of putative strong promoters and their total score. Click here for file
