# Supplementary material for: Triad pattern algorithm for predicting strong promoter candidates in bacterial genomes
Source: BMC Bioinformatics. 2008 May 9;9:233. doi: 10.1186/1471-2105-9-233 (PMC2412878; doi:10.1186/1471-2105-9-233)
Supplement: Additional file 1 — ReadMe. Contains information to use the algorithm. [file 1471-2105-9-233-S1.pdf]

INSTRUCTION
How to work with *the strong_promoter* program?

1. To start the program click on the file ***strong_promoters.doc***. If Word asks on disabling/enabling macros choose to enable macros. If the program does not yet respond, in menu Tools find the option “Macro”, open “Security” and select “Moderate level”.

2. Open the main window FIND_TRIAD (in this version it is SEARCH FOR UP-ELEMENTS):

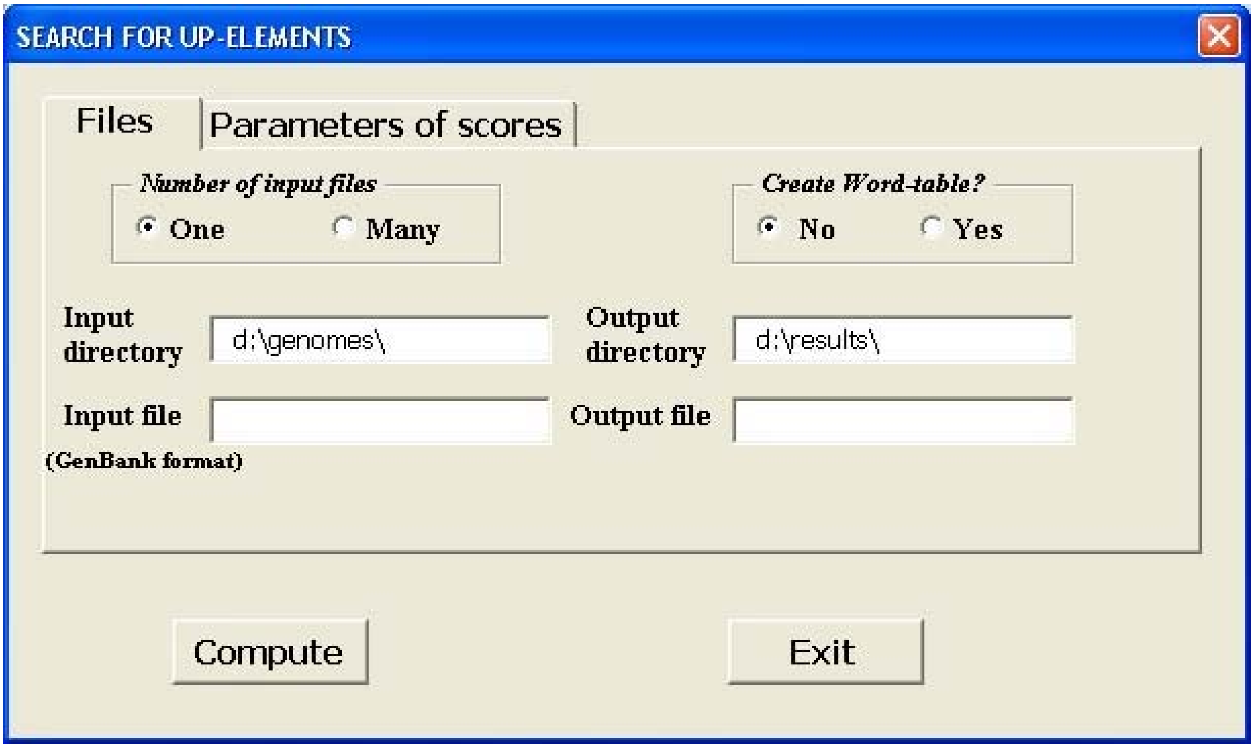

Pic. 1

It includes two pages: **Files** and **Parameters of scores**, and two buttons: **Compute** and **Exit**.

3. The page **Files** has two pairs of radio-buttons: ***Number of input files*** and ***Create Word-table?*** and four small windows to input names of the input and output directories and input file (in the Gen Bank format) and output file (it will have the extension *.txt*) In this version the possibility of multiple input files is disabled. If one choose **Yes** in ***Create Word-table?*** group, then the program creates two output files: one in Text format and the another in the form of Word table. E.g., in the case shown on the pic. 2 the program creates files *ups-ae000512.txt* and *ups-ae000512t.doc* in the output directory *e:\bio\results*.

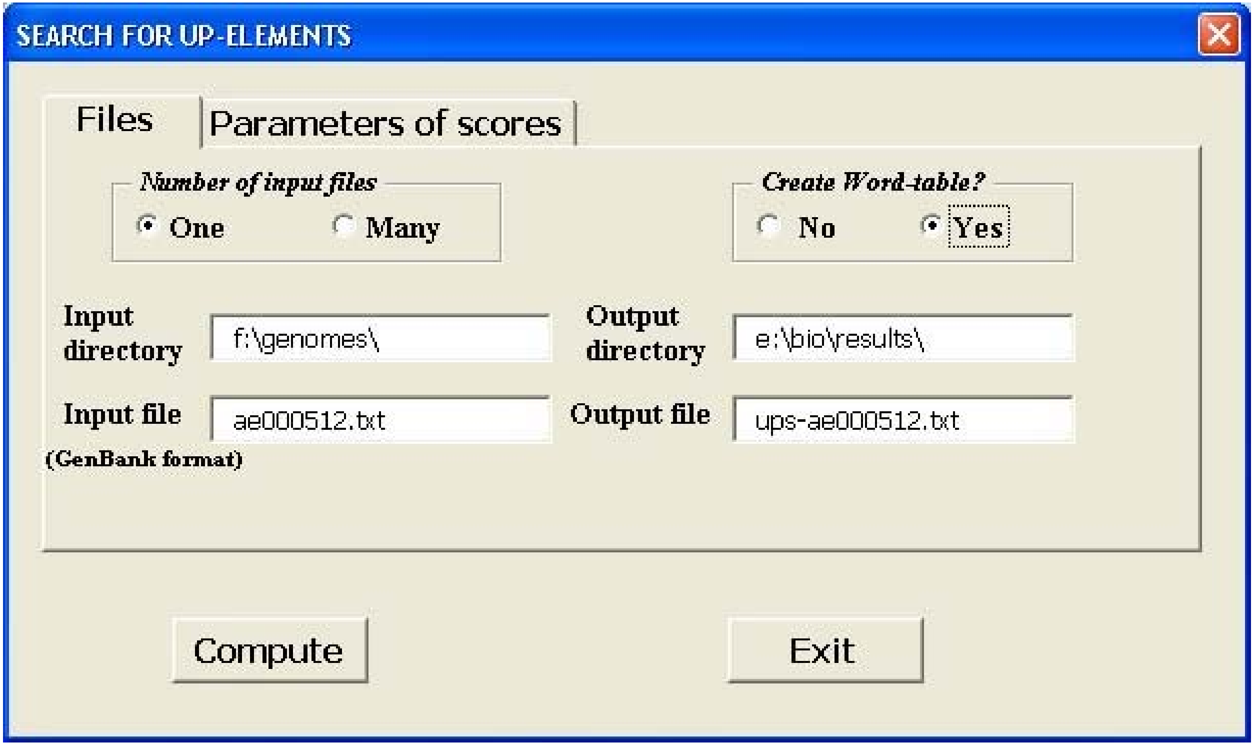

Pic. 2

Caution: *the output directory should exist before starting the program!*

4. The page **Parameters of scores** has three parameters: *scup, sc35* and *sc10* determining the minimal acceptable value of similarity between candidate sequences of the UP-like element, the -35 site, and the -10 site, respectively. Predefined values of these scores are equal to **11** from 17, **5** from 8.5 (the first letter in -35-pattern *tcttgacat* has score 0.5), and **4** (from 6), respectively.

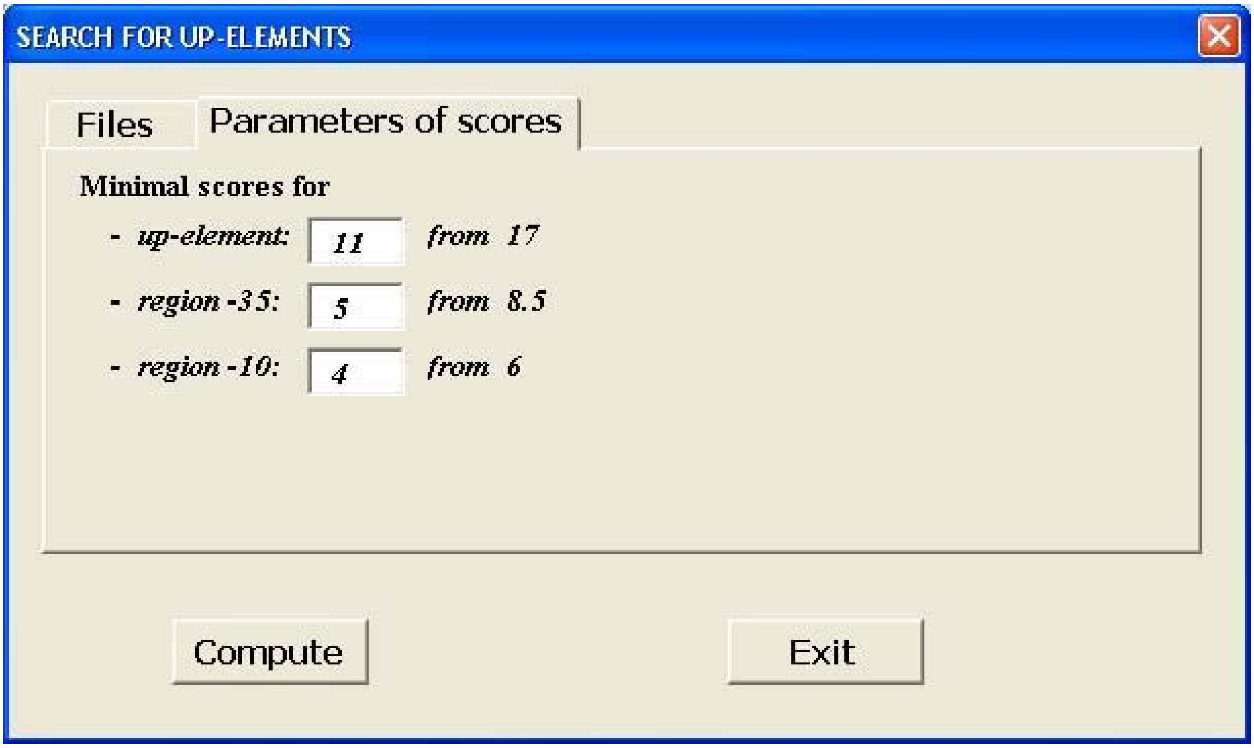

Pic. 3

One can change these predefined values and select other criteria as it is shown on pic. 4.

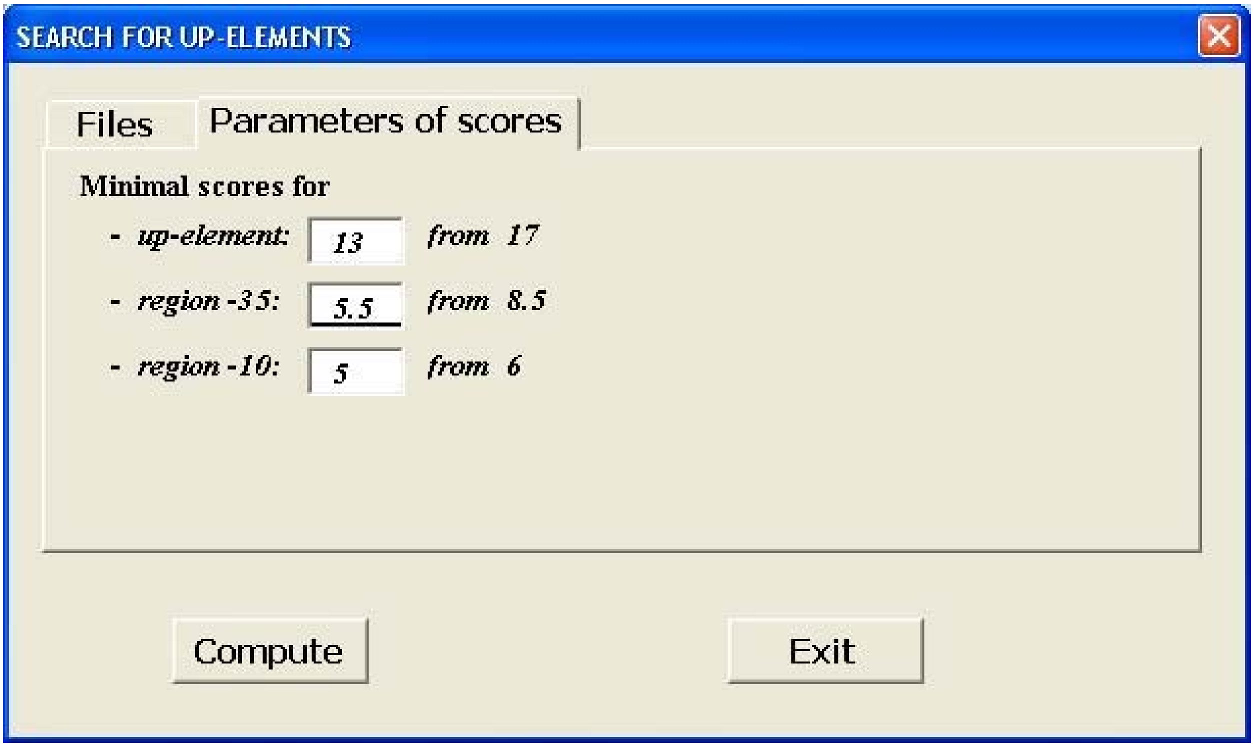

Pic. 4

5. When scores for each consensus are chosen click on **Compute** button to start searching for strong promoters. The computation takes from several seconds to several minutes depending on the computer used. After computing the program shows the message in the form

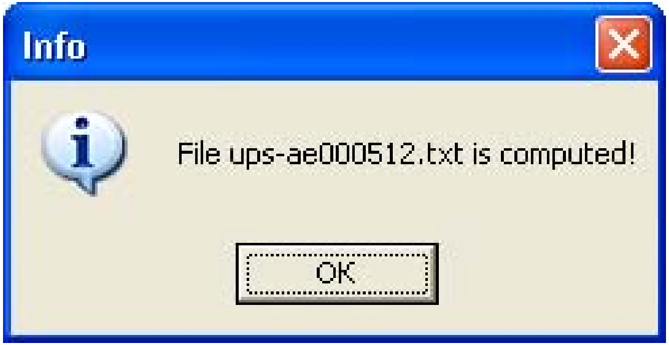

Click on the **OK** button.

6. To finish the process click on **Exit** button.

7. Example. The Text-format of a putative strong promoter "TM1780" predicted in the *T. maritima* genome (***ae000512.txt***) includes:

a) the position of the gene identified, it's name and coded product,
b) the position of the first symbol of UP-like element,
c) the total score of a putative promoter,
d) the individual scores of UP-like element, -35 and -10 sites and a suggested distance between the –35 and -10 sites,
e) the distance between –10 site and the sequence preceding the initiation codon,
f) f) if the promoter is located upstream of an operon then a list of all genes is included.

```
===========================================================================
   gene          1756038..1757267 /gene="TM1780"
        /product="argininosuccinate synthase"
 up_element: 1755953                                total score: 0,875
Scores: up-element                -35 pos.                    -10 pos.
        14                              6     <----------19 --------->   6
AAAATAACAGTGAAAAAacACTTCATATaaatcatttcaaataatccTATAAT
<---- 15 bp ---->aaagaggagggttcatcATG
--------------------------------------------------------------
   gene          1757264..1758459 /gene="TM1781"
   gene          1758456..1759475 /gene="TM1782"
        /product="N-acetyl-gamma-glutamyl-phosphate reductase"
   gene          1759492..1760685 /gene="TM1783"
        /product="glutamate N-acetyltransferase"
   gene          1760682..1761530 /gene="TM1784"
        /product="acetylglutamate kinase"
   gene          1761533..1762690 /gene="TM1785"
        /product="acetylornithine aminotransferase"
^^^^^^^^^^^^^^^^^^^ End of operon (6 genes)^^^^^^^^^^^^^^^^^^^
  ========================================================================
```

File ***ups-ae000512t.doc*** contains a tabulated list of putative strong promoters in the format of Word:

**Downstream gene**	**UP-characteristics / promoter**	**Score**
TM0013 conserved hypothetical protein Operon: 2 genes	ACAATTTTTATCTGATA TTTTCACATtcaccatagtcgatTATAAC <--- 97 bp --->aatctggaggtgacaatATG	0,8475

Note. In the Word format the sequence displayed between putative UP-like element and –35 site might lack some nucleotides as the algorithm permits deletions/insertions during matching (for explanation see the text). Therefore, for further exploitation of the sequence one should use its Text-format as it contains a real genome region.
